# Ecto-domain phosphorylation promotes functional recovery from spinal cord injury

**DOI:** 10.1038/srep04972

**Published:** 2014-05-15

**Authors:** Kenji Suehiro, Yuka Nakamura, Shuai Xu, Youichi Uda, Takafumi Matsumura, Yoshiaki Yamaguchi, Hitoshi Okamura, Toshihide Yamashita, Yoshinori Takei

**Affiliations:** 1 Department of Genomic Drug Discovery Science, Graduate School of Medicine, Osaka University, Osaka, Japan; 2 Department of Molecular Neuroscience, Graduate School of Medicine, Osaka University, Osaka, Japan; 3 Department of Systems Biology, and Graduate School of Medicine, Osaka University, Osaka, Japan; 4 Department of Nanobio Drug Discovery Science, Graduate School of Pharmaceutical Science, Kyoto University, Kyoto, Japan and Graduate School of Medicine, Osaka University, Osaka, Japan

## Abstract

Inhibition of Nogo-66 receptor (NgR) can promote recovery following spinal cord injury. The ecto-domain of NgR can be phosphorylated by protein kinase A (PKA), which blocks activation of the receptor. Here, we found that infusion of PKA plus ATP into the damaged spinal cord can promote recovery of locomotor function. While significant elongation of cortical-spinal axons was not detectable even in the rats showing enhanced recovery, neuronal precursor cells were observed in the region where PKA plus ATP were directly applied. NgR1 was expressed in neural stem/progenitor cells (NSPs) derived from the adult spinal cord. Both an NgR1 antagonist NEP1-40 and ecto-domain phosphorylation of NgR1 promote neuronal cell production of the NSPs, in vitro. Thus, inhibition of NgR1 in NSPs can promote neuronal cell production, which could contribute to the enhanced recovery of locomotor function following infusion of PKA and ATP.

The adult mammalian central nervous system (CNS) cannot be repaired spontaneously after injury. The lack of regenerative capacity in the mammalian CNS is partly due to the myelin-associated proteins including Nogo-A^1^, myelin-associated glycoprotein^2^,^3^ and oligodendrocyte myelin glycoprotein^4^,^5^. Nogo-66 receptor 1 (NgR1) is a common receptor for the myelin-associated inhibitors of the regeneration (i.e., Nogo-A, myelin-associated glycoprotein and oligodendrocyte myelin glycoprotein)^6^, as well as chondroitin sulphate proteoglycans^7^. Inhibition of NgR activation with neutralising antibodies against Nogo-A^8^, a peptide mimicking NgR1-binding region of Nogo-A (NEP1-40)^9^ or the ecto-domain part of NgR1 (NgR1(310) ecto-Fc)^10^, can promote functional recovery of the spinal cord after traumatic injury. Thus, inhibition of NgR activation can promote recovery from spinal cord injury (SCI).

NgR1, along with NgR2 and NgR3, belongs to the NgR family of receptors^11^. Inhibition of NgRs is known to promote neurite sprouting^9^,^12^,^13^ and synapse formation^14^,^15^,^16^,^17^. Triple knockout of NgR1, NgR2 and NgR3, but not single knockout, increases both dendrite outgrowth and synapse number in the mouse hippocampus^18^. NgR1 participates in the postnatal maturation of the CNS^19^,^20^. These reports indicate that NgRs are involved in the regulation of synapse plasticity of the CNS neurons under the physiological conditions.

We previously reported that treatment of cells with protein kinase A (PKA) phosphorylated the ecto-domains of NgR1, NgR2 and NgR3, and that casein kinase 2 phosphorylated NgR1 and NgR2^13^. Phosphorylation of these receptors impedes the binding of the myelin-associated agonists.

Here, we found that administration of PKA and ATP promotes recovery from SCI. NgR1 was expressed in neural stem/progenitor cells (NSPs) derived from the adult spinal cord. Inhibition of NgR1 during in vitro differentiation of the NSPs enhanced neuronal cell production. Consistent with these results, administration of PKA and ATP phosphorylated NgR1 in the spinal cord in vivo and induced cells expressing markers for neuronal precursor cells, which is strictly inhibited without the treatment.

## Results

### Effects of PKA and ATP on the damage from SCI

**Figure 1:**
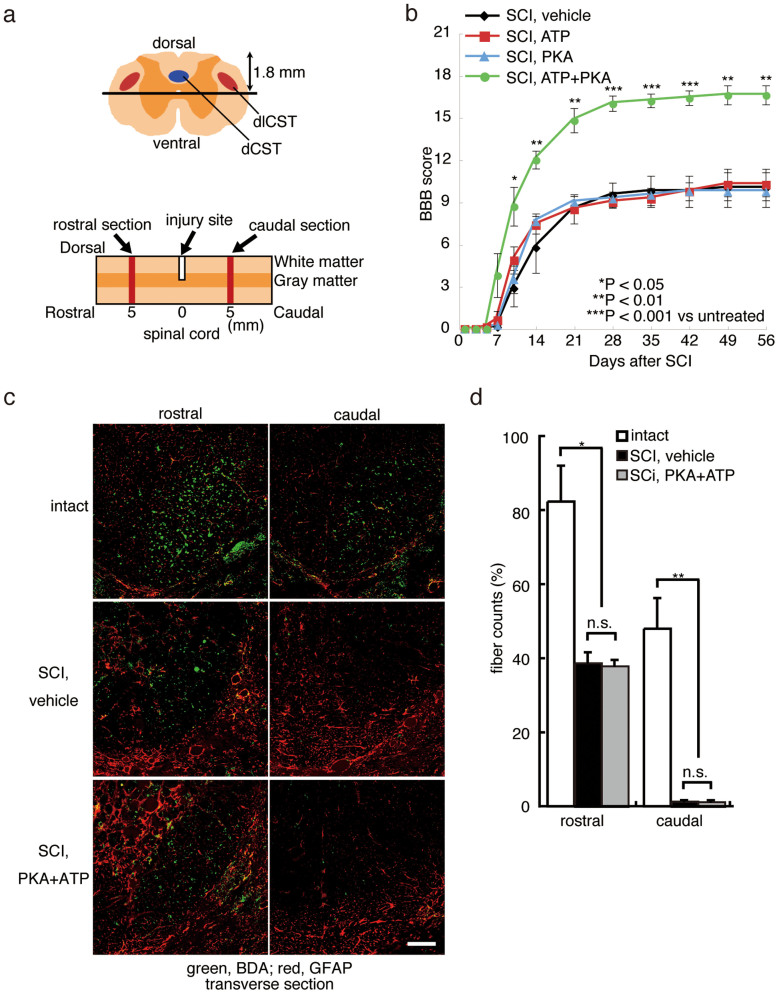
Treatment with PKA plus ATP diminishes damage from traumatic SCI. (a) The depth of injury and location of sections used in (c) are illustrated schematically. The dorsal corticospinal tract (dCST) and the dorsolateral corticospinal tract (dlCST) were severed. (b) The BBB scores of vehicle-treated, PKA-treated, ATP-treated and PKA+ATP-treated SCI rats were assessed at the indicated days after SCI. The points on the graph indicate the average BBB score from six independent rats, and the error bars indicate the standard deviation (S.D.) (*p < 0.05, **p < 0.01, ***p < 0.001 vs. vehicle-treated rats, Student's t-test). (c) The BDA-labelled dCST was visualised. Images are taken from transverse sections at either 5 mm caudal or rostral to the lesion, as shown in (a). The bar indicates 25 µm. (d) The number of BDA-positive axons at T8 or T10 was normalised to the number of BDA-positive axons at C1 (intact region of the spinal cord). The average and the S.D. from three independent animals are shown. No significant differences between the vehicle-treated rats and the PKA/ATP-treated rats were observed (*p < 0.05, **p < 0.01, Student's t-test).

As described schematically in Fig. 1a, we performed a dorsal hemisection on the spinal cords of Wistar rats at the T9 vertebral level. Hind limb locomotor function of the injured rats, both with and without administration of PKA plus ATP, was completely blocked after the induction of SCI (Fig. 1b). From 5 days after SCI, locomotor function gradually improved. By the Day 56 after SCI, the Basso-Beattie-Bresnahan (BBB) score of the injured rats treated with PBS vehicle, PKA alone or ATP alone was less than 10, demonstrating limited spontaneous recovery of locomotor function (Fig. 1b). However, the BBB score of injured rats co-treated with PKA and ATP was significantly augmented relative to the controls at every experimental time point after the Day 10, and it reached to 17 on the Day 56 (Fig. 1b). The hind limbs of the injured rats co-treated with PKA and ATP supported the body weight of the animals (Supplemental Movies). These observations demonstrate that co-treatment with PKA and ATP encourages functional locomotor recovery following traumatic injury to the spinal cord.

To examine elongation of corticospinal axons beyond the lesion epicentre, an anterograde neuronal tracer biotinylated dextran amine (BDA) was injected into the sensorimotor cortex at the Day 42 after SCI. In the absence of SCI, injected BDA stained the corticospinal tract (CST) at the Day 56, as shown in Figure 1c (intact). However, when a lesion was created on the spinal cord at the T9 vertebra, BDA-positive axons were decreased significantly in the sections prepared from 5 mm caudal to the lesion (Fig. 1c, SCI, vehicle). The number of BDA-positive axons was also decreased in the CST 5 mm rostral to the lesion, suggesting the degeneration of axons after SCI. Co-treatment with PKA and ATP failed to increase the number of neuronal fibres stained with BDA, either rostral or caudal to the lesion (Fig. 1c and d). These results indicate that both SCI treated with vehicle and with PKA and ATP received comparable damage on the spinal cord, and that the treatment with PKA and ATP does not promote axonal outgrowth from the sensorimotor cortex.

### Effects of co-treatment with PKA and ATP on neural stem cells

**Figure 2:**
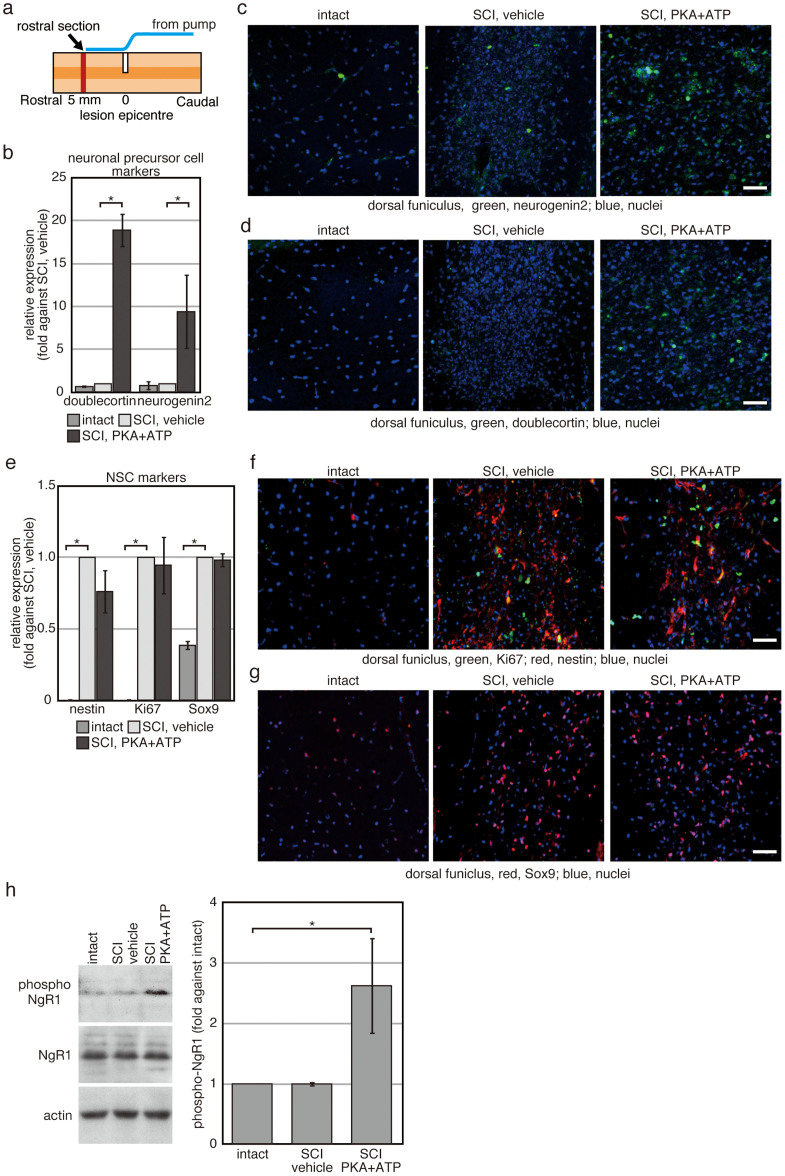
Extracellular treatment with PKA plus ATP increases the number of neuronal precursor cells in the peri-injury site. Rats with SCI were treated with either vehicle or PKA plus ATP for 1 week. (a) Location of sections used in (c, d, f, g) is illustrated schematically. The sections were prepared from the spinal cord directly under the end-point of the tube from the infusion pump. (b–g) Transverse sections prepared from the spinal cord directly under the end-point of the tube from the infusion pump were used for immunofluorescence with indicated antibodies (c, d, f, g). Representative images of the dorsal funiculus were shown. The bars indicate 25 µm. In (b) and (e), signals from indicated antibodies were quantified by using ImageJ 1.48k (available at http://rsb.info.nih.gov/ij/). The average of the three independent animals was plotted and error bars indicate the S.D. (*p < 0.05, Student's t-test). (h) Western blotting analysis of proteins expressed in the spinal nerves. Cell extracts were prepared from the spinal cord at 1 week after SCI, and 40 µg of protein was analysed for each condition. Experiment was repeated for 3 times and intensities of signals were quantifyd by using Image J 1.48k. The average was plotted and error bars indicate the S.D. (*p < 0.05, Student's t-test).

To investigate further the effects of the treatment with PKA and ATP, the spinal cord was dissected on the Day 7 of SCI, when the effects of co-treatment with PKA and ATP began to become evident (Fig. 1b). It was reported that NSPs migrate into the peri-injury site after injury of the CNS^21^,^22^,^23^,^24^. While NSPs can differentiate into both neurons and glial cells in vitro, they preferentially produce glial cells in the peri-injury site in vivo. However, we observed cells expressing markers for neuronal precursor cells, neurogenin 2 (Fig. 2c) and doublecortin (Fig. 2d), only in the tissues co-treated with PKA and ATP. The proportion of cells expressing doublecortin and neurogenin 2 were increased approximately 20-fold and 10-fold, respectively, relative to injured spinal cords exposed to PBS vehicle (Fig. 2b). The location of the cells expressing markers for neuronal precursor cells was limited in the tissues directly under the end-point of the tube from the infusion pump, where PKA plus ATP were directly applied. These results suggest that exposure to PKA plus ATP increases the number of neuronal precursor cells, although the affected region is limited.

The number of cells expressing nestin, a common marker of NSPs, was amplified in the dorsal funiculus after SCI, as was the number of cells expressing Ki67, a marker of dividing cells (Fig. 2e and f). In addition, the number of cells expressing Sox9, a transcription factor expressed in ependymal cell-like and astrocyte-like NSPs, was increased in the dorsal funiculus (Fig. 2e and g). These results indicate that treatment with PKA plus ATP did not affect migration of NSPs into the peri-injury site of SCI.

Western blotting analysis with an anti-NgR1 antibody detected similar levels of NgR1 expression in vehicle-treated spinal cords vs. PKA/ATP-treated spinal cords (Fig. 2h). This demonstrates that overall NgR1 expression was not changed by concomitant exposure to PKA and ATP. Meanwhile, Western blotting with an antibody against phospho-serine^281^ NgR1 revealed a strong signal in PKA/ATP-treated spinal cords. Thus, administration of PKA plus ATP results in the in vivo phosphorylation of ecto-domains of membrane proteins, including NgR1 at serine^281^, which can inhibit agonist-binding to NgR1^13^.

### Expression of NgR1 in NSPs derived from the adult spinal cord

**Figure 3:**
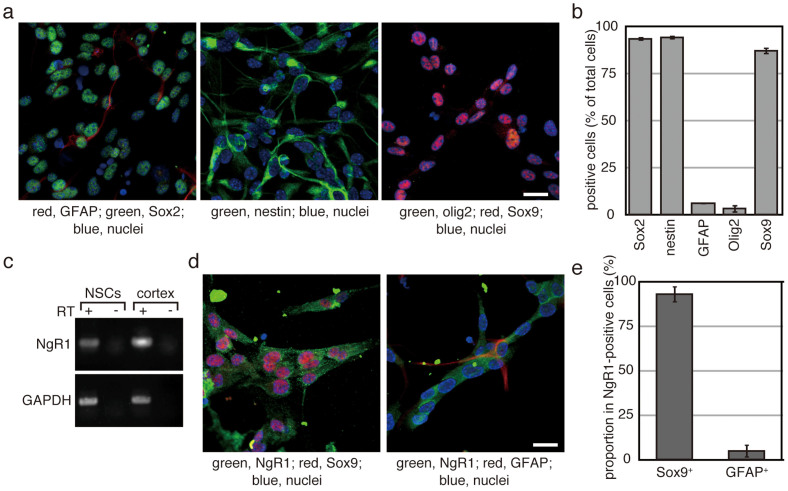
NSPs derived from the adult spinal cord express NgR1. (a and d) After three rounds of neurosphere formation, NSPs were maintained as a monolayer culture. Cells were seeded onto plastic coverslips coated with polyornithine and laminin. After 2 days in culture, cells were fixed and used for immunofluorescence analysis with the indicated antibodies. The bars indicate 50 µm. (b) Cells expressing the indicated proteins were manually counted. DAPI-positive cells were also counted to obtain the total cell number. Each experiment was repeated four times, and more than 200 cells per sample were examined. Each bar and error bar represents the average cell number and the S.D., respectively. (c) Total RNA was prepared from cultured NSPs and used as the template for RT-PCR. Complementary DNA prepared from the adult rat cortex was used as a positive control. Glyceraldehyde 3-phosphate dehydrogenase (GAPDH) was employed as an internal standard. (e) Sox9-expressing cells and GFAP-positive cells were counted in NgR1-positive cells, respectively. They were indicated as percentage against the number of examined NgR1-positive cells. The results were obtained from 3 independent clones of NSPs and their average was shown. Experiments were repeated 3 times. Error bars indicate the S.D.

To ask the relationship between ecto-domain phosphorylation and increased number of neuronal progenitor cells, NSPs were prepared from the adult spinal cord by using a neurosphere culture method and were then maintained as a monolayer culture. The majority of the obtained cells expressed Sox2, nestin and Sox9, but neither glial fibrillary acidic protein (GFAP) nor oligodendrocyte transcription factor 2 (Olig2) (Fig. 3a and b). Several types of cells, oligodendrocyte precursors, astrocytes and ependymal cells, have been reported to be NSPs in the spinal cord^21^,^22^,^25^,^26^. Sox2, as well as nestin, is a common marker for those NSPs. GFAP is a marker for astrocytes. Olig2 is expressed in oligodendrocyte lineage cells and some types of neuronal precursor cells^27^,^28^,^29^. Therefore, expression profile of the obtained cells is similar to that of ependymal cells.

RT-PCR indicated expression of NgR1 in the NSPs obtained from the adult spinal cord (Fig. 3c). Immuno-staining indicated that co-expression of Sox9 and NgR1. More than 90% of NgR1-positive cells expressed Sox9, whereas only less than 5% of NgR1-positive cells expressed GFAP (Fig. 3d and e). Thus, majority of cells expressing NgR1 was Sox9^+^GFAP^−^-cells. These results indicate that NgR1 is expressed in ependymal cell-like NSPs derived from the adult spinal cord.

### Effects of ecto-domain phosphorylation on the neuronal cell production of adult NSPs

To ask the effects of PKA-mediated ecto-domain phosphorylation on neuronal cell production, the NSPs were differentiated by withdrawal of EGF and FGF2 (i.e., no growth factors for the first 2 days of culture, followed by the addition of brain-derived neurotrophic factor (BDNF) from the Day 3 onward).

**Figure 4:**
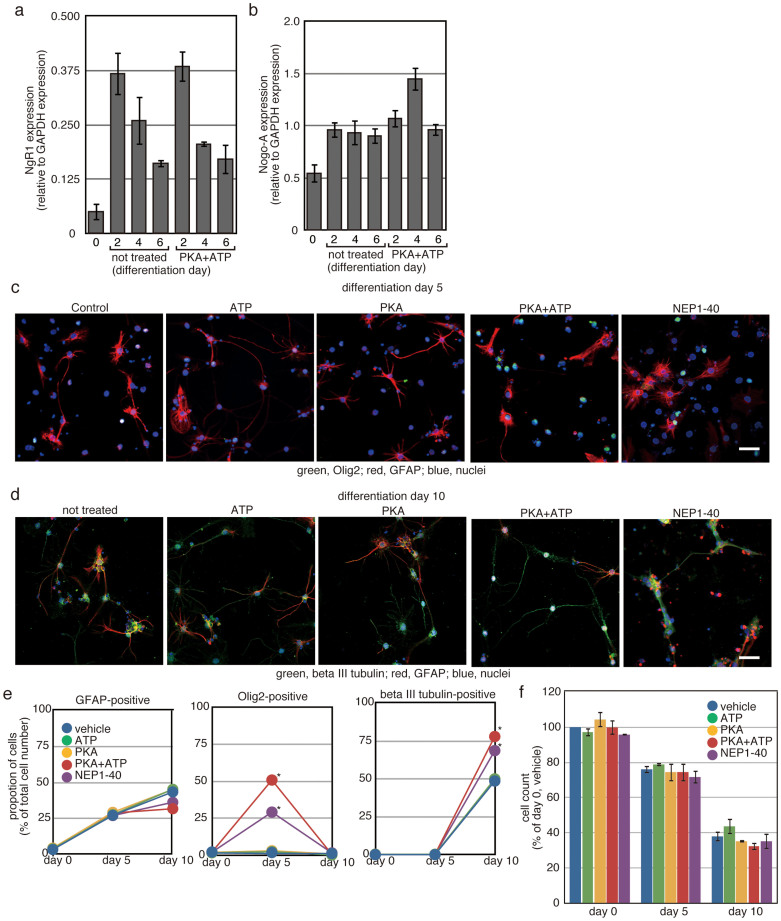
Inhibition of NgR1 enhances neuronal cell production. Differentiation of NSPs derived from the adult spinal cord was induced by depletion of EGF and FGF-2 in the presence of the indicated reagents. (a and b) Total RNA was prepared from the differentiating NSPs on indicated differentiation day, and its cDNA was used as the template for quantitative RT-PCR. Glyceraldehyde 3-phosphate dehydrogenase (GAPDH) was employed as an internal standard. Average of three independent experiments was shown and error bars indicate the S.D. (c and d) On the Day 5 (c) and the Day 10 (d) of differentiation, cells were fixed and used for immunofluorescence analysis with primary antibodies against the indicated proteins. The bars indicate 25 µm. In (e), cells expressing the indicated proteins were manually counted. DAPI-positive cells were also counted to obtain the total cell number. Each experiment was repeated 3 times with 3 independent clones of NSPs, and more than 200 cells per sample were examined. (*p < 0.05 vs. vehicle-treated NSPs, Student's t-test). The bar indicates 25 µm. (f) On the Day 0, 5 and 10 of differentiation, cells were harvested and cell number of each samples were counted, as described in the Methods section. The cell numbers were indicated as percentage against the number of cells treated with vehicle at the Day 0. Each experiment was repeated 3 times with 3 independent clones of NSPs.

After induction of differentiation, NgR1 expression was increased, and treatment with PKA and ATP had no effects on the increase in NgR expression (Fig. 4a). Furthermore, Nogo-A expression was also increased after induction of differentiation (Fig. 4b). When the cells were treated with PKA and ATP, transient increase in Nogo-A expression was observed on the Day 4 of differentiation.

The percentage of GFAP-positive cells increased from about 5% to approximately 30% after 5 days in culture under differentiation conditions (Fig. 4c–e). On the Day 10 of differentiation, the proportion of GFAP-positive cells increased to approximately 40%. Both PKA plus ATP and NEP1-40 tend to decrease the proportion of GFAP-positive cells on the Day 10 of differentiation, although it was not significant.

Proportion of Olig2-positive cells was not changed by 10 days differentiation, and it was less than 5%. Nonetheless, treatment with PKA plus ATP increased the proportion to approximately 50% at the Day 5 of differentiation. NEP1-40 also increased the proportion of Olig2-positive cells. No Olig2-positive cells were detected at the Day 10 of differentiation.

The proportion of beta III tubulin-positive neurons made up less than 1% of the total cell population even on the Day 5 of differentiation, and it was increased to approximately 50% on the Day 10. Treatment with PKA plus ATP increased the proportion of beta III tubulin-positive neurons to more than 75%. NEP1-40 also increased the percentage of beta III tubulin-positive neurons.

Treatment with PKA plus ATP had no effects on the change of the total cell number in the course of differentiation (Fig. 4f). Taken together, these results indicate that PKA-mediated ecto-domain phosphorylation can increase production of neuronal cells from NSPs derived from adult spinal cord, via transient increase in Olig2-positive cells.

### Ecto-domain phosphorylation of NgR1 is required for the increased production of neurons

**Figure 5:**
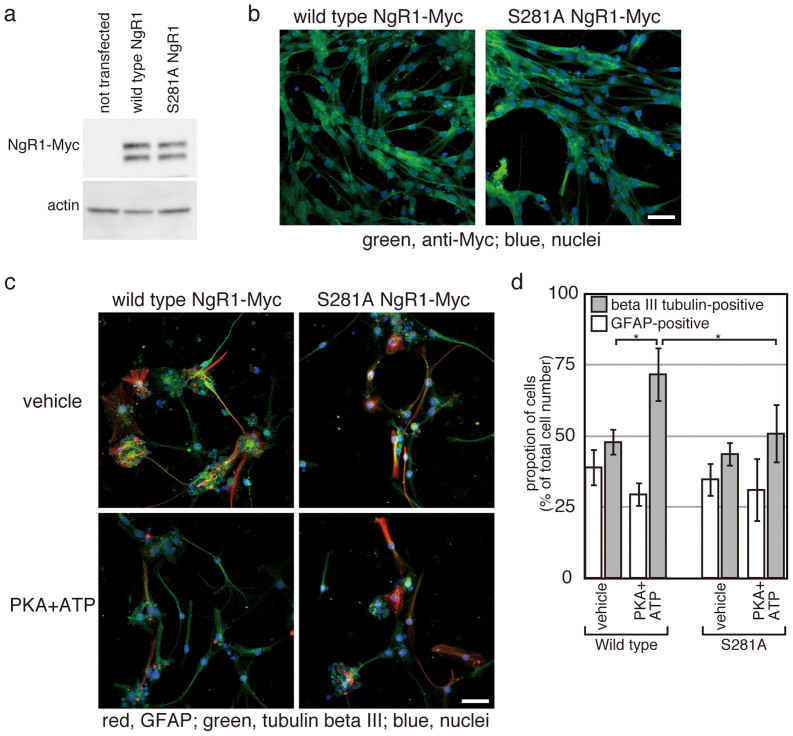
NgR1-phosphorylation is essential for enhancement of neuronal cell production induced by treatment with PKA and ATP. Plasmids encoding either wild type NgR1 or S281A mutant NgR1 were transfected into NSPs before the induction of differentiation. Expression of wild type and mutant NgR1 were examined by Western blotting (a) and immunofluorescence analysis (b). In (a), 40 µg of each protein sample was analysed by SDS-PAGE. Actin was employed as an internal standard. The bars indicate 25 µm. (c) At the Day 10 of differentiation with or without PKA plus ATP, cells were fixed and stained for GFAP, beta III tubulin and DAPI. The bars indicate 25 µm. In (d), cells transfected with either wild type or the S281A mutant NgR1 were treated as in (c) and manually counted. Each experiment was repeated four times, and each bar and error bar represents the average proportion and the S.D., respectively. Differences between the proportions of beta III tubulin-positive cells in the cells transected with mutant NgR1 were not statistically significant. (*p < 0.05, Student's t-test).

Next, we examined the contribution of NgR1 phosphorylation to the increased neuronal cell production. Wild type NgR1 and the non-phosphorylatable S281A NgR1 mutant^13^ were separately overexpressed in the NSPs. Expression levels and transfection efficiencies of wild type and the mutant NgR1 were comparable (Fig. 5a and b). Treatment with PKA plus ATP increased percentage of beta III tubulin-positive cells differentiated from NSPs overexpressing wild type NgR1 (Fig. 5c and d), as the results indicated in Figure 4. However, the treatment had no effects on the percentage of beta III tubulin-positive cells differentiated from NSPs overexpressing the mutant NgR1 (Fig. 5c and d). These results indicate that phosphorylation of NgR1 is essential for promotion of neuronal cell production by co-treatment with PKA and ATP.

## Discussion

The adult CNS shows only poor recovery from traumatic injury, and inhibition of NgR activation has beneficial effects on the recovery^8^,^9^,^10^. Effects of NgR activation are extensively studied with differentiated neurons^30^. However, NgR expression appears not to be limited in the differentiated neurons. Wang, F. and Zhu, Y. reported expression of NgR1 in nestin-positive cells derived from the spinal cord of postnatal day 1 rats^31^. They indicated that an agonist for NgR1 Nogo-p4 can inhibit neurite outgrowth of neuronal cells differentiated from the nestin-positive cells. Wang, B. et al. reported that addition of another NgR1 agonist Nogo66 decreases in neuronal cell production of nestin-positive cells derived from the telencepharon of neonatal rats^32^. Since both papers employed nestin as the only marker for NSPs, types of NSPs expressing NgR1 were unknown. Moreover, since both papers used cells derived from the immature CNS, it was unknown whether NgR1 expression is maintained in NSPs in the adult CNS or not.

This paper indicated expression of NgR1 in ependymal cell-like NSPs derived from the mature spinal cords. Moreover, Nogo-A expression was detected in the differentiating NSPs, and both NEP1-40 and ecto-domain phosphorylation of NgR1 promoted neuronal cell production from the adult NSPs without ectopic addition of NgR1 agonists. These results indicated that NgR1 has a role in the cell fate decision of NSPs, at least under the in vitro conditions. Thus, signalling through NgR1 appears to be able to affect the cells involved in the neural cell lineage from very early stage of neuronal differentiation to terminally differentiated neurons.

During the in vitro differentiation of the adult NSPs, transient increase in the population of Olig2-positive cells was observed on the Day 5 of differentiation of both cells treated with PKA plus ATP and with NEP1-40. Nogo-A expression is also transiently increased on the differentiation Day 4 of cells treated with PKA plus ATP. Since both Olig2 and Nogo-A are expressed in the oligodendrocyte lineage cells, it suggests a possibility that inhibition of NgR1 activation could induce differentiation of NSPs into oligodendrocytes, but the cells could not survive in the in vitro conditions. Since oligodendrocytes can promote survival of neuronal cells^33^,^34^, the transient increase in oligodendrocyte population could contribute to the augmented proportion of neuronal cells observed in the differentiation Day 10. Our results indicated that inhibition of NgR1 in adult NSCs can promote neuronal cell production. However, it is still uncertain whether the increased neuronal cell production is due to promotion of neuronal cell differentiation or survival.

While administration of PKA plus ATP had no effects on the migration of NSPs following SCI, the treatment phosphorylated NgR1 and increased the number of neuronal precursor cells, which is consistent with the in vitro experiments. Although ecto-domain phosphorylation of NgR1 was essential for the augmentation of neuronal cell production *in vitro*, our results do not exclude the possible contribution of ecto-domain phosphorylation of other proteins, *in vivo*, in addition to the ecto-domain phosphorylation of NgR1. These observations suggest that ecto-domain phosphorylation by PKA can allow production of neuronal cells in the adult spinal cord, which is strictly inhibited *in vivo*.

Neuronal progenitor cells in the peri-injury site can be beneficial for regeneration of the injured spinal cord, as indicated by cell transplantation studies^35^,^36^,^37^. While some mechanisms have been proposed for the enhanced recovery, including production of new neurons^38^,^39^ and secretion of growth factors and neurotrophic factors^40^,^41^,^42^,^43^, the detail has not been elucidated yet. It is tempting to assume that ecto-domain phosphorylation of NgR1 expressed in NSPs promotes neuronal cell production, contributing to the augmentation of recovery from SCI. If this is the case, signalling through NgR1 activated after traumatic injury could inhibit regeneration of adult CNS not only through inhibition of synapse plasticity of differentiated neurons but also through inhibition of neuronal cell production of NSPs migrated into the peri-injury site. However, neuronal progenitor cells were observed only in the limited area even after application of PKA plus ATP. Further study is required for assessing the contribution of the neuronal progenitor cells induced by application of PKA plus ATP to the augmentation of recovery from the SCI.

In summary, this study demonstrated that NgR1 is expressed in NSPs derived from adult spinal cord, where its activation can inhibit neuronal cell production at least *in vitro*. Administration of PKA plus ATP phosphorylated NgR1 in vivo, and increased the number of neuronal precursor cells in the limited region of the spinal cord. Furthermore, this treatment promoted functional recovery from SCI. Thus, our results indicated that ecto-domain phosphorylation by PKA could be beneficial for the recovery from SCI.

## Methods

### Antibodies

Anti-Trk A and anti-Myc tag antibodies were purchased from Cell Signaling Inc. Anti-nestin, anti-Sox2 and anti-GFAP were purchased from Merck. Anti-Ki67, anti-Sox9 and anti-doublecortin were purchased from abcam. Anti-neurogenin 2 was purchased from R&D. Anti-NgR1 and anti-beta III tubulin were purchased from Sigma. Anti-phospho NgR1 (serine^281^) primary rabbit antibody was prepared by Cambridge Research Biochemicals Limited (Cambridge, UK).

### Surgical procedures

All surgical procedures and postoperative care were reviewed and approved by the Animal Care and Use Committee of Kyoto University (Kyoto, Japan), and were carried out in accordance with the approved guidelines. Female Wistar rats, 8 weeks of age, were anesthetised by inhalation of 2.0% isoflurane. The skin over the thoracic vertebrae was shaved and cleaned with an iodine tincture. The skin was incised, and the connective and muscle tissues were dissected to expose the lower thoracic spinal cord. A laminectomy was then performed between the 8th and 10th thoracic spinal vertebrae. The dura was cut with a needle, and a dorsolateral hemisection was made with a No. 11 surgical blade at the 9th thoracic spinal vertebral level (T9) to a depth of 1.8 mm. To ensure that the lesion was laterally complete, the surgical blade was passed through the dorsal part of the spinal cord several times.

PKA (New England Biolab) and ATP (Sigma) were diluted to final concentrations of 100 µM and 2.5 U/µl, respectively, in PBS containing 2 mM MgCl_2_ and 100 µM trinitrophenyl (TNP)-ATP (Sigma). TNP-ATP is an inhibitor of P2X receptors, of which activation causes pain. PKA alone, ATP alone or a mixture of PKA plus ATP was applied to the injured region by intrathecal administration via a subcutaneously implanted iPrecio micro infusion pump (Primetech, Ibaraki, Japan). Solutions were administered at a flow rate of 1 µl/h for 14 days. The solution in the pump was changed every 2 days. The pump was implanted subcutaneously at the site of the lesion, and a tube originating from the pump was inserted into the subarachnoid cavity for local administration. The pump was initiated immediately after surgery.

### Locomotor testing

Behavioural analyses were performed to assess open-field locomotor function based on the BBB scale^44^. The BBB open-field test was scored on days 1, 3, 5, 7, 10, 14, 21, 28, 35, 42, 49 and 56 after SCI to evaluate hind limb motor function with regard to stepping and coordination. Each rat was observed for 2 min during each session.

### Immunofluorescence analysis

Anesthetised rats were transcardially perfused with PBS, followed by 4% paraformaldehyde in PBS. The spinal cords were removed, postfixed in the same fixative, and immersed overnight in PBS containing 30% sucrose. The spinal cords were then embedded in Tissue-Tek OCT and kept frozen at −80°C until use. Serial transverse sections (30 μm thick) were cut on a cryostat and mounted on Matsunami adhesive silane-coated Superfrost/Plus slides (Matsunami, Osaka, Japan). The sections were washed three times with PBS and blocked in PBS supplemented with 5% bovine serum albumin (BSA) and 0.1% Triton X-100 for 1 h at room temperature. Immunocytochemistry was performed on the mounted sections with the indicated antibodies. Sections were incubated with primary antibodies overnight at 4°C, followed by the appropriate secondary antibodies for 1 h at 37°C.

For immunostaining of NSPs, cells were washed once with PBS, fixed with 4% paraformaldehyde and 0.5% Triton X100 in PBS overnight at 4°C, and treated as described above.

### BDA tracing

The rats were stabilized in a stereotaxic frame (Narishige) after deep anaesthesia with isoflurane. The scalp was retracted and a circular craniotomy 4 mm in diameter was made with a drill on the right side with the centre at 2 mm antero-posterior, 2 mm lateral to the bregma. An anterograde tracer BDA (10% in PBS) was injected at a depth of 1.5 mm from the cortical surface using a 5 µl micro-syringe tipped with a pulled glass micropipette. After injection, the skin overlying the skull was sutured. Two weeks after BDA injection, the spinal nerves were dissected and sections were prepared as described in “Immunofluorescence”.

For visualisation of BDA, the sections were incubated at room temperature in 5% BSA and 0.1% Triton X-100 in PBS for 1 hour, and then with Alexa Fluor 488-conjugated streptavidin for 2 hours. To quantify the BDA-labelled CST axons in the T8 and T10 vertebra, pixels in the labelled fibres of the dorsal CST were counted for each animal, and this value was divided by the number of pixels in the labelled dorsal CST axons located C1.

### Cell culture

For the preparation of NSPs from the adult spinal cord, a cell suspension was prepared from the spinal cord with Nerve Cell Dissociation Solution (Sumitomo Bakelite Co., Ltd., Tokyo, Japan). The cells were seeded in a soft collagen gel (1 × 10^4^ cells in 35 mm culture dish) and cultured for 3 weeks by using the NeuroCult Neural Colony-Forming Cell Assay kit (Stem Cell Technologies). Independent neurospheres of >2 mm in diameter in collagen gel were harvested separately and disrupted with collagenase and pipetting. Dissociated cells from each neurosheres were cultured separately in serum-free medium containing fibroblast growth factor (FGF)-2 and epidermal growth factor (EGF) for secondary neurosphere formation. After 10 days, floating neurospheres were harvested and treated with Accutase Cell Detachment Solution to prepare a single cell suspension. This step was repeated once. The dissociated cells derived from the neurospheres were cultured in N2B27 medium^45^ containing FGF-2 and EGF on dishes coated with polyornithine and laminin. The medium was changed every day.

For differentiation of NSPs, the cells were seeded on plastic cover slip coated with polyornithine and laminin in a 24-well plate (1 × 10^5^ cells/well), with N2B27 media containing FGF-2 and EGF. Medium was changed to N2B27 without FGF-2 or EGF in the next day (day 0 of differentiation) and cells were cultured for 10 days. From the Day 3 of differentiation onward, BDNF (10 ng/ml) was added to the N2B27 medium.

For cell counting, Accutase Cell Detachment Solution was used for detachment of cells. The cells were washed with Neurobasal medium and resuspended into 500 µl of neurobasal medium. The cell concentration was estimated with a cell counter Scepter 2.0 (Millipore). For transfection of NgR1 plasmids^13^ into NSPs, NeuroMag (Oz Biosciences) was used according to the manufacturer's recommendations.

### Western blotting analysis

One week after SCI, the rats were anesthetised by inhalation of 2.0% isoflurane. The lower thoracic spinal cord was exposed by laminectomy. A portion of the spinal cord between 10 mm rostral and caudal to the lesion epicentre was dissected. The dissected spinal cord was washed with PBS, diced with a surgical blade, and homogenised in a buffer containing 50 mM Tris-HCl (pH 7.8), 150 mM NaCl, 1 mM EDTA, 0.5 mM EGTA, 2 mM DTT, 2× PhosSTOP phosphatase inhibitor mix (Roche) and 1× Complete Protease Inhibitor Cocktail (Roche). After centrifugation at 15,000 × g for 15 min at 4°C, the supernatant was collected as a cell extract. Proteins (40 µg) were analysed by sodium dodecyl sulphate polyacrylamide gel electrophoresis and blotted onto a polyvinylidene difluoride membrane. The membrane was incubated with blocking buffer containing 5% skim milk and 0.5% Triton X-100 in PBS for 1 h at room temperature, followed by the indicated primary antibody in blocking buffer overnight at 4°C and a peroxidase-labelled secondary antibody for 1 h at room temperature. Enhanced chemiluminescence-Plus reagent (GE Healthcare) was used to visualise the bound antibodies.

NSPs shown in Fig. 5 were transfected with either wild type NgR1/pDisplay or S281A mutant NgR1/pDisplay^13^ and cultured overnight in N2B27 medium containing FGF-2 and EGF. Differentiation was induced by depletion of the growth factors. Two days after the induction of differentiation, protein fractions were prepared and used for Western blotting as described above.

### RT-PCR

Total RNA was prepared with Trizol reagent (Invitrogen) from NSPs cultured as a monolayer and used for cDNA synthesis with Superscript II (Invitrogen). Complementary DNA was prepared from 250 ng total RNA and used as a template for RT-PCR with Taq polymerase (Takara Bio Inc., Shiga, Japan). The following sequences were employed for the amplification of NgR1: 5′-CTGGAGGGTAGCAACACCATGC-3′ and 3′-AGTGCAGCCACAGGATAGTGAG-5′. The PCR products were separated by agarose gel electrophoresis, stained with ethidium bromide, and visualised with a UV transilluminator. For quantitative PCR, the following sequences were employed for the amplification of NgR1: 5′-CAGCGAATCTTCCTGCATGGC-3′ and 3′-GTGAAGGCAGCAGCATCGATCC-5′, and for the amplification of Nogo-A: 5′-GTGTTCAGCATTGTCAGTGTAACG-3′ and 3′-GTGGCCTTCATCTGATTTCTGG-5′.

## Author Contributions

K.S., Y.N., S.X., Y.U., M.T. and Y.T. performed the experiments. Y.Y., H.O., T.Y. and Y.T. conceived the experiments. Y.T. wrote the manuscript. All authors discussed the results and commented on the manuscript.

## Supplementary Material

Supplementary Information movie 1

Supplementary Information movie 2

Supplementary Information movie 3

Supplementary Information supplementary information
