# Supplementary material for: Ecto-domain phosphorylation promotes functional recovery from spinal cord injury
Source: Sci Rep. 2014 May 15;4:4972. doi: 10.1038/srep04972 (PMC4021324; doi:10.1038/srep04972)
Supplement: Supplementary Information [file srep04972-s4.pdf]

Supplementary information of “Ecto-domain phosphorylation promotes functional recovery from spinal cord injury”

Kenji Suehiro, Yuka Nakamura, Shuai Xu, Youichi Uda, Takafumi Matsumura, Yoshiaki Yamaguchi, Hitoshi Okamura, Toshihide Yamashita and Yoshinori Takei

Movie S1.Typical behaviour of SCI rats treated with PKA plus ATP, Day 1 after SCI.

Movie S2.Typical behaviour of SCI rats treated with PKA plus ATP, Day 56 after SCI.

Movie S3.Typical behaviour of SCI rats treated with vehicle, Day 56 after SCI.

The SCI rats treated with indicated reagents were placed independently at the centre of an open field (1 m diameter) on the indicated day, and their behaviour was recorded with a video camera.

As shown in Movie S1, hind limb locomotor function was completely blocked at the Day 1 from SCI. After 56 days from SCI, rats treatment with PKA plus ATP show enhanced recovery of hind limb locomotor function, and the hind limbs can

support the body weight of the animals (Movie S2). Although SCI rats treated with vehicle show spontaneous recovery of locomotor function (Movie S3), it was limited as described in Figure 1.

Supplementary figure

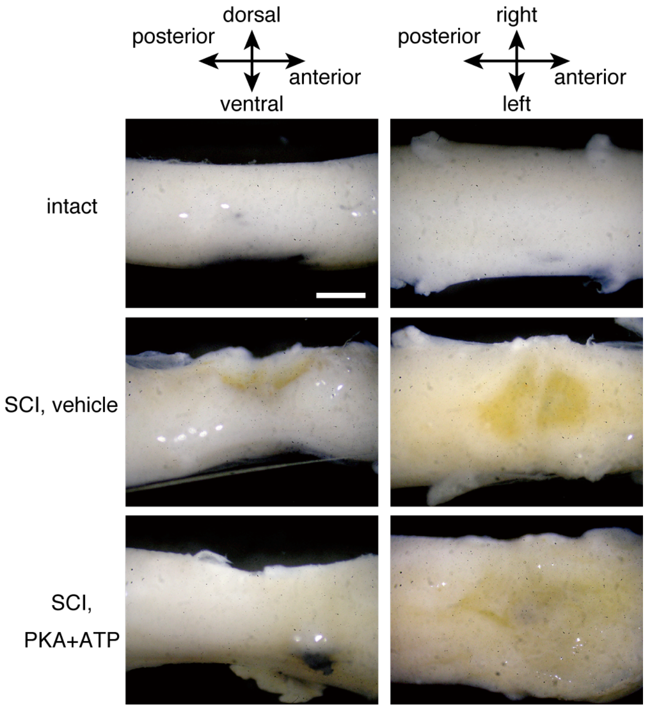

Typical images of the spinal cord at the T9 level prepared from rats with and without SCI. SCI was made as described in the “Surgical Procedures” section. After 1 month from SCI, rats were anaesthetised and transcardially perfused with PBS, followed by 4% paraformaldehyde in PBS. The spinal cords were removed and

postfixed in the same fixative overnight, and then immersed overnight in PBS containing 30% sucrose. The ventral side of the T9 level was marked with black ink. Bar indicates 1 mm. The lesions observed in spinal cords treated with and without PKA+ATP were similar. Tissues were shrunk by fixation.
